# Outcome of Endoscopic Ultrasound-Guided Sampling of Mediastinal Lymphadenopathy

**DOI:** 10.1155/2022/4486241

**Published:** 2022-03-07

**Authors:** Tae Young Park, Jeong Seop Moon

**Affiliations:** ^1^Division of Gastroenterology, Chung-Ang University College of Medicine, Seoul, Republic of Korea; ^2^Department of Internal Medicine, Inje University Seoul Paik Hospital, Seoul, Republic of Korea

## Abstract

**Methods:**

From May 2006 to January 2017, patients with mediastinal lymphadenopathy, who received an EUS-guided trucut biopsy or an FNA biopsy, were retrospectively reviewed. Demographic data, endosonographic characteristics of LNs including size, shape, border, echotexture, and echogenicity, diagnostic yield, and adverse events between the trucut needle group and aspiration needle group were compared.

**Results:**

A total of 69 patients (trucut group, *n* = 33 vs. aspiration group, *n* = 36) were identified. There were no significant differences in demographic data, indication for an EUS-guided biopsy, location of LNs, number of needle passes, and endosonographic features of LNs between the two groups. The sizes of LNs were larger in the trucut group than in the aspiration group (28.9 ± 14.0 mm vs. 21.1 ± 8.8 mm, *P* = 0.007). However, there was no significant difference in the ratio of LNs that were ≥10 mm in both groups. The overall accuracy of the EUS-guided biopsy for the diagnosis of malignant lesions was 79.7% (55/69). There were no significant differences in the histological diagnostic yield of malignant LNs between the two groups. There were no significant procedure-related adverse events in both groups.

**Conclusion:**

The EUS-guided biopsy can be a useful method for histologic evaluation of mediastinal nodal lesions.

## 1. Introduction

Mediastinal lymph nodal staging is the most important consideration when selecting the optimal treatment strategy for lung cancer. In a lung cancer nodal staging work up, endobronchial ultrasound (EBUS)- guided transbronchial needle aspiration is preferred for evaluating mediastinal lymph nodal stages [1, 2] In centers in which EBUS is unavailable, mediastinal lymph node (LN) excision using mediastinoscopy under general anesthesia can be performed. However, excision biopsy using mediastinoscopy can be an expensive and invasive procedure, and it can be associated with a prolonged hospital stay or postoperative complications such as pneumonia and mediastinitis [3]. All centers do not have EBUS equipment, or EBUS is not always available. Therefore, endoscopic ultrasound (EUS)- guided transesophageal fine needle aspiration (FNA) in mediastinal lymphadenopathy is the only alternative method of histologic confirmation without surgical intervention.

In addition, in diseases other than lung cancer that can cause mediastinal lymphadenopathies, including tuberculosis, esophageal cancer, lymphoma, and sarcoidosis, the EUS-guided tissue acquisition of mediastinal LNs is a suboptimal diagnostic method for histologic confirmation that supports a specific treatment including antituberculous therapy and chemotherapy, cytotoxic therapy, or immunosuppressive therapy [4, 5].

Currently, in most of large volume centers where EBUS is available, the EBUS-guided transbronchial mediastinal LN biopsy is a standard method for tissue acquisition of mediastinal LNs. However, in some centers, where EBUS is not available, the EUS-guided transesophageal mediastinal LN fine needle biopsy or aspiration is still used as a suboptimal diagnostic method [6]. This study was aimed at evaluating clinical outcomes of the EUS-guided biopsies of mediastinal lymphadenopathy by comparing a 19-gauge trucut needle group and 22-gauge FNA needle group.

## 2. Methods

### 2.1. Patients

From May 2006 to January 2017, patients with mediastinal lymphadenopathy who received an EUS-guided trucut biopsy or a FNA biopsy at Inje University Seoul Paik Hospital were retrospectively reviewed. This study was approved by the Institutional Review Board of the Inje University Seoul Paik Hospital (IRB No. PAIK 2018-10-005-001).

### 2.2. Endoscopic Procedure

Written informed consent for the EUS-guided tissue acquisition of mediastinal lymphadenopathy was obtained from all of the patients before the procedures. All of the patients underwent EUS-guided trucut biopsy or FNA biopsy in one session. The procedures were performed under conscious sedation in a left lateral position using midazolam (0.05 mg-0.1 mg/kg) and/or propofol (0.5 mg-1 mg/kg). The analgesic agent used was intravenous meperidine (25 mg). To control duodenal peristalsis, intravenous hyoscine-N-butylbromide was routinely administered. Intravenous third-generation cephalosporin was administered prophylactically before the endoscopic procedure. An anterior oblique-viewing linear array echoendoscope (UCT 240; Olympus Optical Co. Ltd., Tokyo, Japan) was used in the procedure. The procedure was performed by one experienced endosonographer (J.H.L.).

Endosonographic characteristics including size (≥10 mm vs. <10 mm), shape (round vs. elongated, ellipsoidal, or triangle), border (sharp demarcated vs. fuzzy), echotexture (homogeneous vs. heterogeneous), and echogenicity (hypoechoic vs. hyperechoic, isoechoic, or mixed echoic) of LNs were evaluated with the B-mode of EUS [7]. After endosonographic evaluation of the mediastinal LNs, tissue sampling from the lesions was performed under EUS guidance. Either a 19-gauge trucut biopsy needle (Quick-Core®, Endoscopic Ultrasound Needle; Cook Endoscopy, Winston-Salem, NC, United States) or a 22-gauge FNA needle (EchoTip® Ultra Endoscopic Ultrasound Needle; Cook Endoscopy) was selected for the procedure at the discretion of the endoscopist (J.H.L.).

### 2.3. Outcomes

The primary outcome was to evaluate the efficacy according to the needle type by comparison of diagnostic yield including accuracy, sensitivity, specificity, positive predictive value (PPV), and negative predictive value (NPV). The secondary outcome was to compare the safety by comparison of procedure-related adverse events including bleeding, perforation, and mediastinitis by needle type.

### 2.4. Statistical Analysis

The categorical variables were compared using a chi-squared test, and the continuous variables were compared using Student's *t*-test. A comparison of accuracy, sensitivity, specificity, PPV, and NPV by needle type was performed with the 95% confidence interval. A *P* value < 0.05 was considered statistically significant. All statistical analysis was performed with IBM SPSS statistics software, version 25.0 (IBM Corp., Armonk, NY).

## 3. Results

A total of 69 patients were identified. The mean age of the study cohort was 63.3 ± 13.6 years, and 51 patients (73.9%) were of male gender. Indications for the EUS-guided biopsies were lung cancer in 52 cases (75.4%), tuberculosis in 6 cases (8.7%), esophageal cancer in 3 cases (4.3%), sarcoidosis in 2 cases (2.9%), lymphangioma in 2 cases (2.9%), lymphoma in 1 case (1.4%), usual interstitial pneumonitis in 1 case (1.4%), atypical pneumonia in 1 case (1.4%), and recurred metastatic lymphadenopathy of stomach cancer in 1 case (1.4%). Locations of LNs were in the subcarinal in 57 cases (82.6%), in the aortopulmonary window in 8 cases (11.6%), in the paratracheal in 3 cases (4.3%), and in the paraesophageal in 1 case (1.4%). The baseline characteristics of the study cohort are summarized in Table 1.

**Figure 1:**
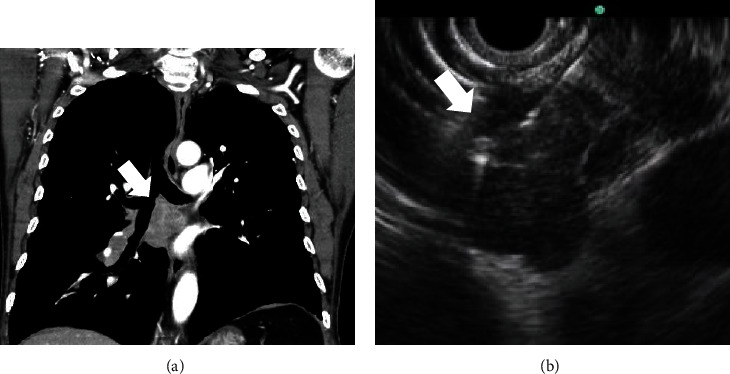
A 78-year-old male patient. (a) Subcarinal lymphadenopathy (arrow) is shown on CT scan. (b) On EUS view, multiple round shaped and sharp-demarcated lymph nodes (arrow) were conglomerated in station 7. The EUS-guided biopsy was performed. CT: computed tomography; EUS: endoscopic ultrasound.

**Figure 2:**
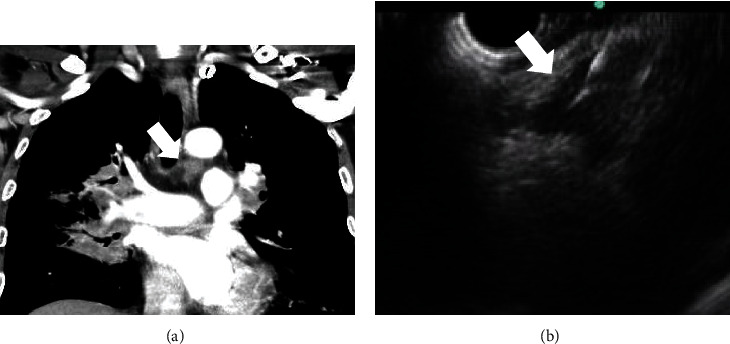
A 53-year-old male patient. (a) Left lower paratracheal lymphadenopathy (arrow) on CT scan. (b) On EUS view, triangle-shaped hypoechoic lymph node (arrow) was observed in station 4L. The EUS-guided biopsy was performed. CT: computed tomography; EUS: endoscopic ultrasound.

Among 69 cases of the EUS-guided mediastinal LN biopsy, 33 cases were performed with a trucut needle and 36 cases were performed with an aspiration needle (trucut group, *n* = 33 vs. aspiration group, *n* = 36). A comparison of characteristics between the two groups is presented in Table 2. There were no significant differences in age, gender, indication of the EUS-guided biopsy, location of LNs, and the number of needle passes between the two groups. The long axes of target LNs were larger in the trucut group than the aspiration group (28.9 ± 14.0 mm vs. 21.1 ± 8.8 mm, *P* = 0.007). The short axis of the LNs was also larger in the trucut group than the aspiration group (15.9 ± 1.1 mm vs. 11.4 ± 5.8 mm, *P* = 0.003). However, there was no significant difference in the ratio of LNs that were ≥10 mm in both groups. Endosonographic features of LNs, including size, shape, border, echotexture, and echogenicity, of the two groups are shown in Table 3. There were no significant differences between two groups in terms of endosonographic features. Two cases of the EUS-guided biopsy of mediastinal lymph node were shown in Figures 1 and 2.

Diagnostic yield for detecting malignant lesions of the total study cohort and comparison by needle type is summarized in Table 4. Overall accuracy of the EUS-guided biopsy for the diagnosis of malignant nodal lesions was 79.7% (55/69). In subgroup comparison, the accuracy, sensitivity, and NPV were higher in the trucut group than the aspiration group. However, there was no statistical significance between the two groups. Specificity and PPV were 100% in both groups. There were no significant procedure-related adverse events in both groups.

## 4. Di*s*cussion

Tissue acquisition of mediastinal LNs is a critical procedure that is performed to determine the treatment plan in lung cancer as well as tuberculosis, esophageal cancer, lymphoma, and sarcoidosis, which can cause mediastinal lymphadenopathies. There have been many invasive techniques for tissue sampling of mediastinal LNs including computed tomography (CT)- guided percutaneous biopsy, mediastinoscopy, mediastinotomy, thoracoscopy, EBUS-guided biopsy, and EUS-guided biopsy [8]. Currently, the EBUS and EBUS-guided biopsies are widely used and considered as essential procedures for the evaluation of mediastinal nodal lesions in lung cancer staging work ups. An EBUS-guided transbronchial approach can be useful for sampling the highest mediastinal (station 1), upper and lower paratracheal (stations 2 and 4), and hilar LNs (station 10), as well as subcarinal LNs (station 7) [3, 8]. However, the EBUS-guided biopsy is not readily available due to regions of LN stations or the absence of a dedicated endoscope for EBUS. Mediastinoscopy is performed under general anesthesia in the operating room. Mediastinoscopy has been demonstrated to be effective and safe in mediastinal nodal staging but can be associated with postoperative morbidity and mortality and high cost, and it may be a cumbersome process. Therefore, the EUS-guided biopsy under conscious sedation can be a minimally invasive, safe, and well-tolerated alternative method to mediastinoscopy. An EUS-guided transesophageal approach can be particularly useful for evaluation of the inferior pulmonary ligament (station 9), esophageal (station 8), subcarinal (station 7), and aortopulmonary window (station 5) LNs, which are difficult to access via mediastinoscopy [3, 8]. Furthermore, the EUS-guided mediastinal lymph node biopsy can be useful for paraesophageal LNs (station 8) or pulmonary ligament LNs (station 9) because common EBUS cannot reach these lymph nodes due to the location.

The EUS-guided FNA was first introduced in 1992 for the evaluation of submucosal or ulcerative lesions of the upper or lower GI tract that are suspicious for malignancy but negative on conventional forceps biopsy [9]. Currently, the EUS-guided FNA has been widely used in pathologic diagnosis of intra-abdominal and mediastinal diseases ranging from malignant lesions to benign lesions [10–15]. Although diagnostic accuracy of the EUS-guided FNA generally has been accepted to be high, the result can be inconclusive. A large amount of tissue is needed for differential diagnosis such as tissue architecture or immunohistochemistry [6]. Sometimes, it is difficult to interpret the results due to blood contamination, necrotic material, or inflammatory cells. Therefore, it would need on-site cytologic evaluation or an additional session of the EUS-guided FNA, and this could be a laborious process. To overcome these limitations, the EUS-guided trucut biopsy using a larger needle caliber was introduced in 2002 [16]. The diagnostic accuracy of the EUS-guided trucut biopsy has been considered to be compatible with those of the EUS-guided FNA [17, 18]. However, because of difficulty of manipulation due to rigidity of the needle and issues of safety due to a spring-loaded firing mechanism, the use of the EUS-guided trucut biopsy has been decreased and practically limited, not only for mediastinal lesions but also pancreaticobiliary lesions [19, 20]. The reverse bevel needle (ProCore® Endoscopic Ultrasound Needle; Cook Endoscopy) was introduced into endoscopic practice, and trucut needle has been replaced into reverse bevel needle. Recently, newer end-cutting FNB needle such as fork-tip design with 2 leading sharp tips (SharkCore™, Medtronic, Minneapolis, Minn) and 3 symmetric cutting edges (Acquire™, Boston Scientific Corp, Natick, Mass) has been also introduced and has been increasingly used [21, 22].

In a most recent study comparing the EUS-guided fine needle biopsy (FNB) vs. EUS-guided FNA in abdominal LN lesions, diagnostic accuracy was significantly higher in the EUS-FNB group as compared to the EUS-FNA group (87.62% versus 75.24%, *P* = 0.02). These findings suggested that EUS-FNB can be preferred to EUS-FNA for sampling intra-abdominal LNs in patients with suspected malignancy [23].

In the current retrospective study conducted at our center, at which EBUS and EBUS-guided mediastinal nodal biopsy are unavailable, the EUS-guided biopsy of mediastinal lymph nodes using a trucut needle or FNA needle is reported. Overall, accuracy of the EUS-guided transesophageal LN biopsy for histologic confirmation was 79.7% (55/69). There were no significant differences in the diagnostic yield by needle type. No significant procedure-related adverse events occurred in both groups.

There are several limitations of note in this study. First, because the EBUS-guided biopsy is unavailable at our center, comparative study regarding this issue could not be performed. Second, trucut needle is commercially unavailable due to safety problem; the findings of this study cannot generally be applied for evaluation of mediastinal lymphadenopathy in clinical practice. Third, current retrospective study was performed using old database in single low volume center. Over the last 10 years, novel FNB needle designs have been developed. 19G Trucut needle was used for acquisition of histological tissue samples for immunohistochemistry at our center. However, 19G trucut needle is not flexible, and its stiffness might render difficulty of sampling in certain situations. Difficulty of manipulation and safety issue limited the use of trucut needle in clinical practice. Nowadays, it has been replaced to ProCore® needle or newer end-cutting FNB needle (SharkCore™ or Acquire™). Fourth, not all patients with mediastinal lymphadenopathy were attempted EUS-guided biopsy. Some patients were initially excluded from the EUS-guided biopsy due to coagulopathy, use of anticoagulant, and difficult location to access by EUS. So, there would be potential of selection bias, and adverse event would be underestimated. Therefore, a randomized, controlled trial in large volume multicenter is needed to assess the efficacy and safety of EUS and EBUS in the future.

In conclusion, the EUS-guided fine needle biopsy can be a useful method for pathologic confirmation of mediastinal nodal lesions, particularly at centers in which EBUS-guided biopsy is unavailable.

## Figures and Tables

**Table 1:** Baseline characteristics of the study cohort (*n* = 69).

Characteristics	Value
Age (y), mean ± SD	63.3 ± 13.6
Male gender, *n* (%)	51 (73.9)
Indications of procedure, *n* (%)	
Lung cancer	52 (75.4)
Tuberculosis	6 (8.7)
Esophageal cancer	3 (4.3)
Sarcoidosis	2 (2.9)
Lymphangioma	2 (2.9)
Lymphoma	1 (1.4)
UIP	1 (1.4)
Atypical pneumonia	1 (1.4)
Recurred metastatic LAP of stomach cancer	1 (1.4)
Location of lymph nodes, *n* (%)	
Subcarinal	57 (82.6)
Aortopulmonary window	8 (11.6)
Paratracheal	3 (4.3)
Paraesophageal	1 (1.4)
Size of lymph nodes (mm), mean ± SD	
Long axis	24.8 ± 12.1
Short axis	13.6 ± 6.5

SD: standard deviation; UIP: usual interstitial pneumonitis; LAP: lymphadenopathy.

**Table 2:** Characteristics by needle type.

	Trucut (*n* = 33)	Aspiration (*n* = 36)	*P* value
Age, mean ± SD	60.7 ± 14.3	65.6 ± 12.6	0.132
Gender, *n* (%)			0.523
Male	24 (72.7)	27 (75.0)	
Female	9 (27.3)	9 (25.0)	
Indication of biopsy, *n* (%)			0.784
Lung caner	22 (66.7)	30 (83.3)	
Tuberculosis	4 (12.1)	2 (5.6)	
Esophageal cancer	2 (6.1)	1 (2.8)	
Lymphoma	1 (3.0)	0	
Sarcoidosis	1 (3.0)	1 (2.8)	
Others^∗^	3 (9.1)	2 (5.6)	
Size of lymph node (mm), mean ± SD			
Long axis	28.9 ± 14.0	21.1 ± 8.8	0.007
Short axis	15.9 ± 1.1	11.4 ± 5.8	0.003
Location of lymph node, *n* (%)			0.829
Subcarinal	26 (78.8)	31 (86.1)	
Aortopulmonary window	4 (12.1)	4 (11.1)	
Paratracheal	2 (6.1)	1 (2.8)	
Paraesophageal	1 (3.0)	0	
Number of needle pass, *n* (%)			0.796
1	24 (72.7)	27 (75.0)	
2	4 (12.1)	3 (8.3)	
3	3 (9.1)	4 (11.3)	
4	1 (3.0)	2 (5.6)	
5	1 (3.0)	0	

^∗^Lymphangioma (*n* = 2), usual interstitial pneumonitis (*n* = 1), pneumonia (*n* = 1), and recurred metastatic lymphadenopathy of stomach cancer (*n* = 1).

**Table 3:** Endosonographic feature of lymph nodes.

	Trucut (*n* = 33)	Aspiration (*n* = 36)	*P* value
Size, n (%)			0.335
≥10 mm	33 (100)	35 (97.2)	
<10 mm	0	1 (2.8)	
Shape, *n* (%)			0.214
Round	19 (57.6)	16 (44.4)	
Elongated or ellipsoidal	10 (30.3)	18 (50.0)	
Triangle	4 (12.1)	2 (5.6)	
Border, *n* (%)			0.453
Sharp demarcated	28 (84.8)	28 (77.8)	
Fuzzy	5 (15.2)	8 (22.2)	
Echotexture, *n* (%)			0.528
Homogeneous	19 (57.6)	18 (50.0)	
Heterogeneous	14 (42.4)	18 (50.0)	
Echogenicity, *n* (%)			0.110
Hypoechoic	16 (48.5)	12 (33.3)	
Mixed echoic	12 (36.4)	10 (27.8)	
Isoechoic	3 (9.1)	12 (33.3)	
Hyperechoic	2 (6.1)	2 (5.6)	

**Table 4:** Diagnostic yield for histologic confirmation by needle type.

	Total (*n* = 69)	Trucut (*n* = 33) [95% CI]	Aspiration (*n* = 36) [95% CI]	*P* value
Accuracy, % (*n*)	79.7 (55/69)	84.8 (28/33) [67.4-100]	75.0 (27/36) [47.5-100]	0.310
Sensitivity, % (*n*)	57.6 (19/33)	66.7 (10/15) [64.8-100]	50.0 (9/18) [39.9-100]	0.335
Specificity, % (*n*)	100 (36/36)	100 (18/18) [NA]	100 (18/18) [NA]	—
PPV, % (*n*)	100 (19/19)	100 (10/10) [NA]	100 (9/9) [NA]	—
NPV, %, (*n*)	78.0 (36/50)	78.3 (18/23) [64.5-100]	66.7 (18/27) [46.7-100]	0.363

CI: confidence interval; PPV: positive predictive value; NPV: negative predictive value, NA: not available.

## Data Availability

The [DATA TYPE] data used to support the findings of this study are available from the corresponding author upon request.

## Abbreviations

EBUS: Endobronchial ultrasound
LN: Lymph node
EUS: Endoscopic ultrasound
FNA: Fine needle aspiration
PPV: Positive predictive value
NPV: Negative predictive value
CT: Computed tomography.
